# The study of total hip arthroplasty outcomes among statin users in Sina Hospital, Iran

**DOI:** 10.1097/MD.0000000000042074

**Published:** 2025-04-04

**Authors:** Erfan Barootchi, Sina Arefi, Pardis Zamani, Mohammad Soleimani, Amir Hossein Karimi, Reza Hajebi, Seyyed Hossein Shafiei

**Affiliations:** a Orthopedic Subspecialty Research Center (OSRC), Sina Hospital, Tehran University of Medical Sciences, Tehran, Iran; b Department of General Surgery, School of Medicine, Sina Hospital, Tehran University of Medical Sciences, Tehran, Iran.

**Keywords:** complications, functional outcomes, statins, total hip arthroplasty

## Abstract

Total hip arthroplasty (THA) is a highly effective intervention for hip osteoarthritis. The increasing demand for THA is driven by an expanding pool of eligible patients, including elderly individuals with comorbidities. Statins, which are widely used to reduce cardiovascular risk, have shown potential benefits in reducing postoperative complications after joint arthroplasty. This study aims to evaluate the influence of statin use on THA outcomes and postoperative quality of life (QOL). This retrospective observational cohort study was conducted at a single center. Patients who underwent primary THA between 2015 and 2021 were identified and categorized into the statin-exposed and non-statin cohorts. Patients were interviewed regarding postoperative complications and completed the Short Form-8 questionnaire to assess their QOL. Statistical analyses were performed using *t* tests, Chi-square tests, and correlation tests. In total, 364 patients were included in the final sample with 37 patients in the statin-exposed cohort. The statin-exposed cohort included significantly older patients with higher comorbidity rates. However, there were no significant differences in postoperative joint or systemic complications or readmission rates between the 2 cohorts. The in-hospital mortality was lower in the statin cohort, but the difference was not statistically significant (0% vs 8.9%, *P* = .059). There was no significant difference between the 2 cohorts based on the Short Form-8 questionnaire (21.65 ± 8.14 vs 20.16 ± 8.67, *P* = .310). Contrary to previous studies, this study did not find significant differences in THA outcomes and QOL between statin-exposed and non-statin cohorts. This disparity may be a result of differences in study design, patient populations, and the timing of statin initiation relative to surgery. The findings of this study should be validated through larger studies to assess the effects of statin use more firmly.

## 1. Introduction

Total hip arthroplasty (THA) is widely recognized as the most effective intervention for moderate to severe hip osteoarthritis.^[1]^ It is predicted that there will be a considerable rise in THA surgery, with an estimated 174% increase by 2030 in the United States.^[2]^ One of the contributing factors for the increasing demand for THA is the expanding pool of patients who are eligible for the procedure. Patients are now undergoing elective hip arthroplasty procedures even in their 90s, thanks to advancements in modern medicine that have extended overall life expectancy.^[3,4]^ As the number of elderly patients undergoing THA continues to rise, it is plausible that they may present with multiple comorbidities and be prescribed a variety of medications. Notably, cardiovascular disease represents a significant cause of morbidity and mortality in older adults worldwide.^[5]^ It is observed that the incidence of cardiovascular events escalates after 65 years in men and after 75 years in women.^[5]^

The effectiveness of statins in lowering the risk of cardiovascular events has been firmly established. In patients over 65 years of age, statin therapy reduces the incidence of major cardiovascular events by 19%, which is comparable to the 22% risk reduction observed in patients under 65 years of age.^[5]^ Multiple studies have shown the ability of statins to reduce postoperative arrhythmia rates after total joint arthroplasty (TJA).^[6,7]^ In addition to their role in reducing cardiovascular events, statins have been shown to directly affect bone cells, suppressing osteoclastogenesis and promoting osteogenesis.^[8]^ For instance, Sax et al reported that preoperative statin exposure reduces periprosthetic fractures and revisions following total knee arthroplasty (TKA).^[9]^ Similarly, Sutton et al showed that statin exposure among United States veterans resulted in significantly lower risk of prosthetic joint infection.^[10]^

With the increasing utilization of THA and a rising trend of THA patients receiving statin therapy, it is crucial to investigate the potential impact of statin exposure on THA outcomes. Therefore, the primary objective of this study is to evaluate the influence of statin use on THA outcomes and quality of life (QOL) following surgery.

## 2. Method

### 2.1. Study design and population

A retrospective observational cohort study was conducted at a single center. The study received approval from the Ethics Committee of Tehran University of Medical Sciences (Reg No. 1401, 142, TUMS.MEDICINE.REC) and all research procedures were carried out in full compliance with its stipulations and the 1965 Declaration of Helsinki, as revised in Tokyo in 2004. Patients aged 18 years or older who underwent primary THA at Sina University Hospital, Tehran, Iran, between January 2015 and January 2021 were retrospectively identified through the electronic health record database. Exclusion criteria were applied to eliminate patients who had a history of cancer, chronic conditions (e.g., chronic kidney disease, chronic obstructive pulmonary disease, chronic liver failure), were currently undergoing radiotherapy or chemotherapy, and those who had undergone revision THA. Following the application of exclusion criteria, the remaining patients were allocated into 2 distinct cohorts based on their use of statins. Subsequently, the patients were contacted and interviewed after their THA surgery. The patients data were gathered through the hospital information system about the occurrence of revision surgery, dislocation, infection, and periprosthetic fracture, as well as any subsequent hospital readmissions following their primary THA. Furthermore, patients were asked to complete the Short Form-8 (SF-8) questionnaire to provide insight into their QOL post-surgery.

### 2.2. The utilized questionnaire

The SF-8 is a validated, abbreviated version of the Short Form-36 Health Survey, which comprises 36 items aimed at measuring the overall health status of individuals. The SF-8 consists of 8 domains, including physical functioning, role physical, bodily pain, general health, vitality, social functioning, mental health, and role emotional. These domains have been designed to provide psychometrically sound summary measures of both physical and mental health. The SF-8 was specifically developed for use in large surveys of general and specific populations, allowing for the comparison of disease burden across different age, disease, and treatment groups. Furthermore, the SF-8 has been demonstrated to be an effective tool for monitoring population health and large-scale outcomes studies.

### 2.3. Outcomes

The outcomes evaluated were cardiac complications (cardiac arrhythmia, cardiac ischemia), acute kidney injury, thromboembolic (deep vein thrombosis/pulmonary embolism), wound infection, in-hospital mortality, periprosthetic fracture, dislocation, revision, and readmission which their data were gathered through hospital information system and QOL score through the SF-8 questionnaire. These variables were also compared independently.

### 2.4. Statistical analysis

Quantitative variables were presented as mean and standard deviation (mean ± SD), while categorical variables were expressed as a percentage. Comparison between quantitative variables was done by *t* test or Mann–Whitney test. Comparison between qualitative variables was also done using the Chi-square test. Pearson or Spearman correlation test was used to evaluate the correlation between quantitative variables. SPSS version 23 software was used for statistical analysis of data. Statistical significance was determined at a *P*-value of <.05. The data supporting this analysis will be provided upon reasonable request from the corresponding author. Subgroup analyses stratified by age, statin dosage and time, and comorbidities were not performed due to sample size limitations. In future analyses, adjustments for confounders such as age, comorbidities, and baseline differences are essential to enhance inferential accuracy (response to comment 4).

## 3. Results

After applying all inclusion and exclusion criteria, in total, 364 patients who underwent THA were included in the final sample (Table 1) with 37 patients in the statin-exposed cohort. The non-statin cohort consisted of 54% males, while the statin cohort had 37.8% males. The statin-exposed patients were significantly older (63.00 ± 12.98 vs 50.25 ± 16.90, *P* < .001) and had a significantly higher incidence of cerebrovascular disease (2.7% vs 1.9%, *P* = .004), diabetes mellitus (29.7% vs 6.1%, *P* < .001), kidney disease (5.4% vs 2.4%, *P* < .001), thyroid disease (16.2% vs 4.7%, *P* = .001), and hypertension (32.4% vs 16.8%, *P* = .026) compared to the non-statin cohort. All other baseline characteristics, including body mass index, marital status, education level, and mean operation time, were similar between the 2 cohorts.

**Table 1:** Baseline characteristics in patients undergoing THA with and without history of statin use.

Characteristics	Without statin (N = 327)	With statin (N = 37)	*P*-value
Male gender, %	176 (54.0)	14 (37.8)	.062
Mean age, year	50.25 ± 16.90	63.00 ± 12.98	<.001
Mean BMI, kg/m^2^	29.57 ± 2.62	27.63 ± 4.35	.753
Marital status, %			.100
Single	48 (22.2)	4 (11.1)	
Married	152 (70.4)	25 (69.4)	
Divorced	2 (0.9)	0 (0.0)	
Widow	14 (6.5)	7 (19.4)	
Education level, %			.194
Illiterate/primary	36 (16.7)	12 (32.4)	
Secondary	94 (43.7)	16 (43.2)	
Diploma	67 (31.2)	8 (21.6)	
Academic degree	18 (8.4)	1 (2.7)	
History of smoking, %	58 (27.0)	7 (19.4)	.340
History of alcohol, %	26 (12.1)	3 (8.3)	.514
History of heart failure, %	5 (2.3)	2 (5.4)	.276
History of peripheral vascular disease, %	5 (2.3)	1 (2.7)	.893
History of cerebrovascular disease, %	4 (1.9)	3 (8.3)	.004
History of pulmonary disease, %	6 (2.8)	1 (2.7)	.973
History of rheumatic disease, %	20 (6.1)	3 (8.1)	.546
History of liver disease, %	6 (2.8)	1 (2.7)	.916
History of diabetes mellitus, %	13 (6.1)	11 (29.7)	<.001
History of kidney disease, %	8 (2.4)	2 (5.4)	<.001
History of thyroid disease, %	10 (4.7)	6 (16.2)	.001
History of hypertension, %	36 (16.8)	12 (32.4)	.026
Mean operation time, hour	3.47 ± 0.74	3.54 ± 0.74	.712

BMI = body mass index, THA = total hip arthroplasty.

No significant difference was observed between the 2 cohorts in terms of postoperative joint complications, such as joint infection, periprosthetic fracture, dislocation, revision rate, and readmission rate (Table 2). Similarly, postoperative systemic complications were similar between the 2 cohorts. However, in-hospital mortality was lower in the statin cohort compared to the non-statin cohort, although this difference was not statistically significant (0% vs 8.9%, *P* = .059). Moreover, there was no significant difference between the 2 cohorts based on the SF-8 QOL questionnaire (21.65 ± 8.14 vs 20.16 ± 8.67, *P* = .310).

**Table 2:** Postoperative outcome in patients undergoing THA with and without history of statin use.

Characteristics	Without statin	With statin	*P*-value
In-hospital mortality, %	29 (8.9)	0 (0.0)	.059
Local infection, %	17 (8.0)	4 (11.1)	.599
Periprosthetic fracture, %	19 (8.9)	4 (11.1)	.236
Dislocation, %	15 (7.1)	4 (11.1)	.599
Revision, %	29 (13.6)	8 (22.2)	.175
Readmission, %	51 (23.6)	8 (22.2)	.889
Mean quality of life score			
Physical component score	11.77 ± 4.42	11.37 ± 5.04	.619
Mental component score	9.87 ± 4.64	8.78 ± 4.47	.186
Total quality of life score	21.65 ± 8.14	20.16 ± 8.67	.310
Mean total length of hospital stay, day	6.38 ± 3.97	7.36 ± 4.29	.169
Mean postoperative hospital stay, day	3.64 ± 3.06	3.94 ± 2.69	.569
Postoperative events, %			
Cardiac ischemic event	8 (4.1)	3 (8.1)	.297
Deep vein thrombosis	5 (2.6)	1 (2.7)	.965
Acute kidney disease	2 (1.0)	0 (0.0)	.999
Pulmonary emboli	3 (1.5)	0 (0.0)	.999
Cardiac arrhythmia	16 (8.2)	5 (13.5)	.307
Brain stroke	4 (2.1)	1 (2.7)	.586
Mean serum creatinine, mg/dL	1.00 ± 0.35	1.10 ± 0.83	.585

THA = total hip arthroplasty.

## 4. Discussion

The use of statin therapy to manage cardiovascular health has been on the rise in the United States, with over 35 million people being prescribed this medication.^[11]^ Besides reducing the risk of cardiovascular events, statins have also been observed to affect bone cells, which may impact THA outcomes. This study seeks to assess the impact of statin use on the outcomes of THA surgery and patients’ QOL after the procedure. This study was carried out at a single center, which limits the generalizability of the findings. Outcomes may vary across diverse populations and healthcare systems due to differences in demographics, surgical techniques, and healthcare practices; therefore, multi-center studies with larger and more diverse populations are required to confirm these results and enhance external validity (according to comment 1). We were able to demonstrate that the statin-exposed cohort were significantly older and had a higher incidence of several comorbidities compared to the non-statin cohort. However, there was no significant difference in postoperative joint complications or systemic complications, and the QOL following surgery was similar between the 2 cohorts. The in-hospital mortality rate was lower in the statin cohort, but the difference was not statistically significant.

The results of our study contradicts the available literature. Bonano et al, reported that only 0.4% of patients who received a perioperative statin developed cardiac arrhythmias compared to 4.0% who did not receive statins after TJA which suggests a 10-fold reduction in the relative risk.^[6]^ Similarly, Chen et al, reported significantly lower rates of cardiac arrhythmias at 2-weeks, 30 days and 90 days following THA.^[7]^ Moreover, multiple studies have reported statins protective effects on prosthetic joint infection, revision and periprosthetic fractures following TKA and THA.^[9,10,12]^ However, such differences were not seen in our patient population.

The difference seen between our study and the available literature might be associated with both the dosage and the timeline of statin used. Studies have shown that it takes a year to see the benefits of statin use.^[5]^ Cook et al reported that participants exposed to statins for more than 5 years following THA/TKA (vs < 1 year) had a reduced risk of revision.^[13]^ The findings of this study contrast with prior researches indicating statins protective effects on bone health, prosthetic joint infections, and cardiac complications. This disparity may be a result of differences in study design, patient populations, and the timing of statin initiation relative to surgery. For example, Cook et al reported significant benefits with long-term statin use, suggesting that our study’s shorter exposure period may have masked potential benefits (response to comment 5). Nonetheless, most studies on the impact of statins in TJA outcomes do not account for the amount and duration of statin use. Future research should consider these factors, as well as whether statin use was preoperative or postoperative, to determine the true impact of statins in TJA outcomes.

Furthermore, the impact of statins on bone metabolism remains uncertain due to contradictory findings. Laboratory studies indicate that statins reduce bone resorption by inhibiting osteoclasts through mevalonic acid reduction.^[14]^ However, randomized controlled trials have failed to validate the potential benefits of statins in preventing fractures.^[15]^ Although some observational studies have suggested that statins could be beneficial in preventing fractures,^[16]^ others have found no evidence of such an association.^[17]^ These contradictory results imply that there may be confounding factors that can enhance or limit the true effect of statins on bone metabolism.

To our knowledge, this is the first study to evaluate the impact of statin use on patients’ QOL following THA. Although our study did not find a significant difference in QOL between the statin and non-statin cohorts based on the SF-8 form, it is important to note that the statin cohort had more health conditions and was likely sicker than the non-statin cohort. Chronic illnesses can have a significant impact on physical, mental, and social functions, leading to increased expenses, isolation, disability, pain, and emotional distress.^[18–20]^ Thus, it is possible that patients in the statin cohort had a lower QOL prior to surgery, and the similar QOL post-surgery suggests that statin use may lead to a greater improvement in QOL. Future studies should evaluate the impact of statin use on patient-reported outcome measures, such as SF-8, to confirm this possibility.

## 5. Limitations

This study has several limitations that must be acknowledged. Primarily, study cohorts were assessed using medical and billing codes, which are susceptible to misclassification and incorrect billing. Secondly, data on the dosage, duration, and timing of statin use were not available, which is a critical limitation as these factors may significantly influence outcomes. Thirdly, our study cohort of only 37 patients who received statins limits the generalizability of our findings. Finally, we were unable to evaluate certain patient, social, and surgical factors, such as race, ethnicity, discharge status, operative time, surgical volume, preoperative functional status, type of prosthesis, and surgical techniques which may have influenced our results. Despite these limitations, our study provides evidence that based on statistical analysis, statin therapy has no significant effect on THA outcomes and QOL; however, future studies should account for these variables to provide a more comprehensive analysis of the effects of statin use on THA outcomes (response to comment 3).

## 6. Conclusion

This study showed that patients in the statin-exposed cohort were older and had a higher prevalence of comorbidities compared to the non-statin cohort. However, there were no significant differences observed in postoperative joint complications, systemic complications, or QOL between the 2 cohorts. These findings contradict previous studies that have reported benefits of statin therapy in reducing cardiac complications and improving outcomes following joint arthroplasty. On account of the relatively small sample size in the statin-exposed cohort, the study’s limited statistical power and the ability to detect significant differences provides hypothesis-generating insights into the impact of statin use on THA outcomes. These findings should be interpreted with caution and validated through larger studies to assess the effects of statin use more firmly (response to comment 2). Future studies are suggested to employ more robust statistical models, including regression analysis, to control for potential confounders and identify causative links (response to comment 4).

## Author contributions

**Data curation:** Erfan Barootchi, Sina Arefi, Pardis Zamani.

**Formal analysis:** Erfan Barootchi, Sina Arefi.

**Methodology:** Mohammad Soleimani, Pardis Zamani.

**Supervision:** Reza Hajebi, Seyyed Hossein Shafiei.

**Writing – original draft:** Erfan Barootchi, Sina Arefi, Pardis Zamani.

**Writing – review & editing:** Erfan Barootchi, Mohammad Soleimani, Amir Hossein Karimi, Seyyed Hossein Shafiei.
